# Prediction of Asthma Exacerbations in Children

**DOI:** 10.3390/jpm14010020

**Published:** 2023-12-22

**Authors:** Evangelia Sarikloglou, Sotirios Fouzas, Emmanouil Paraskakis

**Affiliations:** 1 Medical School, Democritus University of Thrace, 68100 Alexandroupolis, Greece; evsarikloglou@gmail.com; 2 Department of Pediatrics, University of Patras Medical School, 26504 Patras, Greece; sfouzas@upatras.gr; 3 Paediatric Respiratory Unit, Paediatric Department, University of Crete, 71500 Heraklion, Greece

**Keywords:** asthma attack, asthma exacerbation, children, biomarkers

## Abstract

Asthma exacerbations are common in asthmatic children, even among those with good disease control. Asthma attacks result in the children and their parents missing school and work days; limit the patient’s social and physical activities; and lead to emergency department visits, hospital admissions, or even fatal events. Thus, the prompt identification of asthmatic children at risk for exacerbation is crucial, as it may allow for proactive measures that could prevent these episodes. Children prone to asthma exacerbation are a heterogeneous group; various demographic factors such as younger age, ethnic group, low family income, clinical parameters (history of an exacerbation in the past 12 months, poor asthma control, poor adherence to treatment, comorbidities), Th2 inflammation, and environmental exposures (pollutants, stress, viral and bacterial pathogens) determine the risk of a future exacerbation and should be carefully considered. This paper aims to review the existing evidence regarding the predictors of asthma exacerbations in children and offer practical monitoring guidance for promptly recognizing patients at risk.

## 1. Introduction

Asthma is the most common chronic disorder of childhood and represents a significant health burden. The disease is characterized by chronic airway inflammation and acute episodes (exacerbations) of reversible airway obstruction with respiratory symptoms, such as wheezing, dyspnea, chest tightness, and coughing. Currently, asthma monitoring relies solely on the regular assessment of respiratory symptoms and lung function. However, the lack of a direct measurement of inflammation may result in the inappropriate recognition of children at risk for a future asthma attack [1,2,3,4]. 

Asthma exacerbations are not rare in asthmatic children, even among those with apparently reasonable disease control. Asthma attacks result in the children and their parents missing school and work days; limit the patient’s social and physical activities; and lead to emergency department visits, hospital admissions, or even fatal events [5,6,7,8,9,10,11,12]. Thus, the prompt identification of asthmatic children at risk for exacerbation is crucial, as it may allow for proactive measures that could prevent these episodes [13]. Current asthma treatment strategies have generally succeeded in controlling daily symptoms and provide to asthmatic children a good quality of life [8]. Nevertheless, it is estimated that in the United States, half of the children with asthma will experience at least one exacerbation per year, while in Europe, more than one in three will have an unplanned hospital visit due to an asthma attack [14].

Of note, children prone to asthma exacerbation are a heterogeneous group [15]; various demographic factors such as younger age, ethnic group, low family income, clinical parameters (history of an exacerbation in the past 12 months, poor asthma control, poor adherence to treatment, comorbidities), Th2-type of inflammation, and environmental exposures (pollutants, stress, viral and bacterial pathogens) determine the risk of a future exacerbation and should be carefully considered [7,16,17,18,19].

This paper aims to review the existing evidence regarding the predictors of asthma exacerbations in children and offer practical monitoring guidance for promptly recognizing patients at risk.

## 2. Level of Asthma Control

Poor asthma control is a standard risk indicator for asthma exacerbation [12,20,21,22,23]. For example, children with partially controlled asthma have a 2-fold increase in the exacerbation rates, as compared to those with controlled disease [23]. However, symptom-based tools used to assess asthma control, such as the Asthma Control Test (ACT) and the Asthma Control Questionnaire (ACQ), cannot offer precise predictions on the time of exacerbation. In the study by Schatz et al., a lower ACT score was associated with an increased risk of emergency department visits and oral corticosteroid and beta-agonist use in the following 12 months [22,24]. Conversely, in another study by Meltzer et al., each 1-point increase in the ACQ score was associated with a 50% increase in exacerbation risk for the following two weeks [25]. On the other hand, other studies have demonstrated that asthma exacerbations can also occur in the context of reasonable asthma control [10,11,26]. These reports have questioned the predictive utility of ACT and ACQ scores, demonstrating that they are not superior to the frequency of rescue inhaler use alone [10,11,26]. In a 4-year study by Wu et al., 14% of the participants never reported troublesome asthma symptoms, although they had presented at least one severe exacerbation [12]. In another study of 612 asthmatic children, 54% of those who reported good asthma control had abnormal spirometry and or raised fractional exhaled nitric oxide (FeNO) [27]. Clearly, the factors that are associated with poor asthma control are not the same as those associated with asthma exacerbations. Moreover, the loss of disease control may be hard to identify by the patients and their parents [28,29]. Socioeconomic status also plays a crucial role in the way patients perceive and report their symptoms; in a cross-sectional study (N = 307) by Ganti et al., there was a significant positive correlation (*p* < 0.001) between the ACT score and the education and socioeconomic status of the family [30]. Nevertheless, it is generally accepted that ACT and ACQ scores can be used as part of the routine evaluation of asthmatic children and for assessing the risk of future exacerbations [31]. The combination of asthma symptom scores and medication scores could improve our ability to identify children at risk of an asthma attack in the future.

Several studies have shown that an asthma attack is by itself a strong predictor of an exacerbation of the disease in the future. The use of oral corticosteroids and emergency department visits or hospitalizations for symptoms related to asthma in the previous 12 months are strong and independent predictors of a future attack [9,12,18,23,32,33,34,35,36,37,38]. A study by Engelkes et al. demonstrated that patients with an asthma exacerbation have a 25% possibility of repeating the episode within the following year [32]. In a recent systematic review, Lowden et al. confirmed that a past exacerbation is the best predictor of a future exacerbation, regardless of the severity of the disease and the level of control [38]. The number of previous exacerbations is also important [17,37]; in the metropolitan area of St. Louis, the probability of hospital readmission for an asthma exacerbation over ten years increased by 30% after the first admission, 46% after a second admission, and 59% after a third admission [17]. On the other hand, Lowden et al. concluded that the severity of an asthma exacerbation does not necessarily relate to the severity of the previous exacerbations [38]. In any case, the asthma management plan for a given patient should be carefully reviewed when an exacerbation occurs. Various factors, such as female sex, higher FeNO levels, and escalating treatment, are associated with a higher exacerbation risk and, thus, may highlight the need for a more frequent follow-up [39].

## 3. Lung Function Testing

Spirometry is widely used for assessing the lung function of asthmatic patients. However, the test is notoriously unable to detect abnormalities at the level of small airways, as in the case of asthmatic children, where small airways are affected early in the course of the disease [40]. Thus, the existing evidence on the usefulness of spirometry in detecting children at increased risk for asthma exacerbations is conflicting [11,33,41,42]. In a retrospective study of 13,842 children (100,292 observations) seen annually over 15 years, a strong association was noted between FEV1% predicted and risk of asthma exacerbation in the subsequent year [41]. Repeated measurements of FEV1, even if they are within the normal range, could add to the clinical risk assessment; a 10% reduction in FEV1% predicted within three months is associated with 28% increased odds for an asthma exacerbation [43]. Reversibility to bronchodilators may reveal specific obstruction phenotypes, also related to the risk of an asthma attack [44]. In other studies, mid-expiratory flows presented a good predictive value for a future exacerbation, even when the baseline FEV1 was normal [45,46].

Specific peak expiratory flow (PEF) patterns may also be related to loss of asthma control and risk of exacerbation [47]. Wide diurnal PEF variations signify loss of disease control, while a steep PEF decline without changes in variability is observed during exacerbations. Studies using complex statistical methods have suggested that PEF variability could help predict future asthma attacks in adults [48], but similar data in children are lacking. In a study by Kim et al., PEF was lower in asthmatic children in autumn than in winter, suggesting that seasonal variations should also be considered [49].

## 4. Adherence to Treatment

Poor adherence to treatment, including improper inhaled medication and/or breathing chamber use techniques, is associated with an increased risk of exacerbations, hospital admissions, and asthma-related deaths [50,51,52]. Children with asthma who have regular follow-up visits present a reduced risk of asthma attacks, while inadequate follow-up adherence relates to increased morbidity and more frequent exacerbations [53]. Additionally, incorporating patient preferences into treatment decisions (e.g., type of inhaler device, medication dosage) seems to result in longer exacerbation-free periods, especially for children with poor asthma control [54,55]. Interestingly, a recent study showed that during the COVID-19 pandemic, asthma exacerbations were reduced due to decreased exposure to environmental triggers and increased patient adherence [56]. It should be mentioned, however, that other studies failed to confirm a significant impact of adherence to treatment on impending asthma exacerbation in children [57,58].

## 5. Other Patient-Related Factors

A study conducted in the United States showed that race and ethnicity play an important role in adverse asthma outcomes since non-Hispanic black children had a greater risk for emergency department visits and deaths due to asthma compared to their non-Hispanic white counterparts [59,60]. Others have shown that Asian ethnicity is associated with a lower likelihood of future asthma attacks [61], while African American race and low socioeconomic status may increase the risk of asthma exacerbations [62,63]. However, further studies are required to explore the exact role of the genetic background in such populations [64]. Various socioeconomic factors determining the ease of accessing healthcare resources may contribute equally to an increased risk of asthma exacerbations [8,65,66]. Moreover, all these factors may vary and, thus, play different roles according to the child’s age [13].

Overweight or obesity reduces the response to inhaled corticosteroids and predisposes one to asthma attacks. The role of chronic stress and anxiety is more complex and poorly understood, although an increased Th2 cytokine response has been reported [67,68]. In addition, chronic stress may lead asthmatic patients to poor adherence [69,70,71,72,73,74]. Interestingly, maternal depression is also associated with an increased risk of asthma exacerbation in children [75].

## 6. Salbutamol Overuse

A higher number of days of salbutamol use (>two days in two weeks) and a higher number of salbutamol doses per day are strong and independent predictors of severe asthma exacerbation in the future [76]. Short-acting beta-agonist (SABA) overuse has also been associated with an increased risk of death due to asthma [77]. Patients who have learned to “control” their disease only by SABAs need special attention because SABA overuse seems to increase bronchial hyperreactivity and induce pro-inflammatory pathways [76,78,79]. In this regard, the monitoring of SABA use could offer better disease control and prevent future exacerbations. In a study from Sweden, one-third of asthmatic patients (12–45 years old) used three or more SABA canisters per year, while the risk of asthma exacerbation was directly related to the amount of SABA used [80]. In another study, Frey et al. have suggested that the frequent administration of SABAs (>4 times per day) may increase the risk of asthma attacks due to the loss of beta-agonist effectiveness. The prescription of more than three SABA canisters per year should alert healthcare professionals to the risk of an imminent asthma exacerbation [48,77].

Long-acting beta-agonists (LABAs) are more effective in stabilizing airway tone in the long term [48]. However, LABA monotherapy may also be associated with severe asthma exacerbations and asthma-related death, especially in younger children [81]. Nevertheless, the concurrent administration of LABAs with inhaled corticosteroids (ICSs), usually as a fixed LABA-ICS combination, has been associated with the reduced rate and severity of exacerbations and better clinical outcomes than using ICSs alone [81].

## 7. Biomarkers

Airway inflammation biomarkers are constantly evaluated concerning their ability to identify Th2 inflammation. The essential role of the Th2 type of inflammation in asthma exacerbation emerges from clinical trials of “biological” agents, such as IgE, IL-4, IL-5, and IL-13 inhibitors [82]. The administration of these novel drugs has consistently been associated with a significant reduction in asthma exacerbations, thus highlighting the pivotal role of Th2 inflammation in the susceptibility to asthma attacks [82]. Novel technologies that can be applied to multiple biological samples, such as metabolomics, proteomics, transcriptomics, and genomics, hold particular promise for identifying patients with poor disease control and are at risk for asthma exacerbations [82,83]. Among these techniques, “breathomics” is of particular interest due to its non-invasive nature that offers the possibility of frequent and repeated sampling.

Evidence on the utility of FeNO as a predictor of asthma exacerbations in children and adolescents remains conflicting. FeNO, alone or in combination with other biomarkers, is an essential tool for monitoring adherence and response to treatment [84,85,86]. In a recent observational study, Lo et al. correlated FeNO measurements with future asthma exacerbations and showed that higher FeNO levels could predict future asthma attacks [61]. A FeNO of ≥80 ppb has been proven useful in identifying poorly controlled asthma in children [87]. In a small study of adults, those who experienced an asthma exacerbation had significantly higher FeNO levels within two weeks before the event [88]. Moreover, the investigators showed that FeNO was the only significant and independent predictor of exacerbations compared to spirometric indices, quality of life scores, and medication usage [88]. On the other hand, similar studies in asthmatic children found that a single FeNO measurement is not useful in assessing the risk of an upcoming exacerbation [89,90]. In the Reducing Asthma Attacks in Children using Exhaled Nitric Oxide trial, a combined approach based on symptom-guided asthma treatment and FeNO levels did not reduce the asthma attacks [91,92]. Even FeNO measurements two weeks before an exacerbation in children with severe asthma may have poor positive predictive value [93]. In another cohort study from Ecuador, 283 children with asthma were followed for six months or until their next asthma attack; a previous severe exacerbation was the most reliable predictor of a future asthma attack, while the predictive ability of FeNO measurements was limited [94]. FeNO levels in children aged 0–4 years correlate well with the Asthma Predictive Index but cannot reliably predict a future asthma diagnosis or disease exacerbations [95]. A recent study confirmed the low predictive value of FeNO measurements even when combined with clinical characteristics [96], while Fielding et al. demonstrated that a significant increase in FeNO levels between subsequent visits was associated with poor asthma outcomes but not a higher exacerbation risk [43]. On the other hand, two recent meta-analyses concluded that when FeNO is used to guide asthma management strategies, the frequency of asthma exacerbations can be reduced [97,98]. The significant intrasubject variability in FeNO values in children may have accounted for the above controversial findings [99].

FeNO partitioning, i.e., the measurement of FeNO at multiple exhalation flow rates, offers valuable information on the NO concentration in the most distal airways, the so-called alveolar NO (CalvNO) [100]. A recent study from our group explored the role of CalvNO as a predictor for asthma exacerbations in 68 asthmatic children [101]. We found that CalvNO levels > 7 ppb could predict asthma exacerbations in the subsequent four months with 90.9% specificity, while a CalvNO of <4 ppb could exclude a future exacerbation with 71.4% sensitivity. Moreover, an increase in CalvNO by 0.5 ppb between subsequent visits could predict future exacerbations with 92% sensitivity and 92% specificity, while the performance of ACT scores and spirometric indices (including reversibility testing) was significantly lower [101]. Therefore, distal inflammation plays a pivotal role in asthma exacerbations in children and should be further considered in future studies [101].

Sputum eosinophils is a cost-effective biomarker for assessing disease control in asthmatic patients [102]. However, sputum collection may be challenging in young and uncooperative children, while sputum eosinophils do not seem reliable in predicting future asthma attacks [90,93,103]. Novel saliva biomarkers, such as eotaxin, IL-5, and IL-8, are easier to collect and have shown a strong correlation with the level of asthma control [104], but their role is still to be determined.

Measurements of volatile organic compounds (VOCs) in the exhaled breath seems also promising, as specific VOC patterns are closely related to disease exacerbations in asthmatic children [105,106,107].

Generally, blood eosinophil counts (EOSs) of >300 cells/µL have been related to troublesome asthma in adults. In the Severe Asthma Research Program, EOSs > 400 cells/mL were associated with an increased risk of exacerbation [108,109]. However, in asthmatic children, the evidence is conflicting [110,111,112]. EOSs, combined with FeNO, have been used as markers of the Th2 inflammation pathway to predict the response to treatment in asthmatic children, with reasonable results [113,114,115].

Serum IL-6 was also associated with the risk of asthma exacerbation in children, but further studies are required [116]. Plasma eosinophilic cationic protein (ECP) concentration is a useful marker of Th2 inflammation and may help identify children at risk for recurrent asthma attacks who could benefit from corticosteroid treatment [117]. Other biomarkers of atopy, such as skin prick or specific IgE testing for sensitization to aeroallergens and total serum IgE, have been utilized to assess the risk of seasonal exacerbations [118]. Mucosa-associated lymphoid tissue translocation protein 1 (MALT1) is another novel biomarker [119].

Urinary leukotriene E4 (ULTE4) levels reflect systemic cysteinyl leukotriene production [120,121], and, when elevated, may predict asthma exacerbations in children exposed to tobacco smoke [122]. Also, urinary phthalate metabolites and urinary organic acids seem to be significantly associated with imminent asthma attacks [123,124]. More inflammatory mediators, including cytokines, chemokines, IL-5, and acidity levels, can be measured in the exhaled breath condensate and serve as metabolomic biomarkers of asthma exacerbation in the future [36,96].

Studies based on genome-wide association have revealed the existence of susceptibility variants that are specifically related to exacerbations and differ from those generally related to asthma. A cadherin-related family member gene variant (CDHR-3) has been linked to recurrent severe asthma exacerbations in preschool children of European descent [125], while the 17q21 locus and the ADRB2 gene (especially its Glu27 variant) are consistently associated with asthma attacks in asthmatic children and adults [5,126]. A recent meta-analysis demonstrated a significant association between a single-nucleotide polymorphism in FLJ22447 (rs2253681) and severe asthma exacerbations [127]. Furthermore, three microRNA models (miR-146b, miR-206, and miR-720) that could predict exacerbations in asthmatic patients receiving inhaled corticosteroids have been detected [128]. Reduced responsiveness to SABAs, especially in those using long-acting beta-agonists (LABAs), has been associated with polymorphisms in the beta-2 adrenoceptor gene [129]. Finally, nasal airway transcriptomic analysis demonstrated that higher baseline Th2/Th1-interferon ratios can predict asthma attacks [130]. Future studies should explore the full spectrum of such genetic variabilities, with larger sample sizes, better representation of racial/ethnic diversity, and a more precise definition of asthma exacerbation.

## 8. Environmental Exposures

Environmental exposures, including aeroallergens, viral and bacterial pathogens, environmental pollutants, and stress, largely drive asthma exacerbations [131]. Atopic individuals, in particular, have the most significant risk when they are exposed to the aeroallergen to which they are sensitized [132]. The association between viral respiratory tract infection and asthma exacerbations is well established in childhood [133]. For example, in a study by Murray et al., this association tremendously increased the likelihood of an asthma exacerbation [134,135,136]. Such patients remain vulnerable to asthma attacks during respiratory infections even if the level of disease control is good [47]. Human rhinovirus (HRV) infection seems to be the most significant trigger of asthma exacerbations in children, and as such, it might be used as a “biomarker” for imminent asthma attacks. A study from Germany before the COVID-19 pandemic demonstrated that 41% of the children who experienced an exacerbation had a positive test result for HRV, while 14% were positive for the respiratory syncytial virus (RSV) [137,138]. Interestingly, HRV was particularly prevalent among asthmatic and atopic patients (56% and 66%, respectively) [137,138]. Respiratory microbiota and specific bacteria–host interactions may also determine the risk of asthma exacerbations. Several Moraxella and Haemophilus members may enrich viral respiratory illnesses during the fall season, leading to subsequent exacerbations [139]. These episodes seem to have a regular peak after returning to school from their summer holidays, i.e., in September for the Northern hemisphere and in January for the Southern [135,140,141,142].

Asthma exacerbations also present a second peak around the end of the hay fever season [143]. A 10-year-long study from Italy demonstrated that asthma exacerbations had seasonal peaks during autumn and spring. Pollens; wind speed; rainfall; and SO_2_, NO, O_3_, and NO_2_ levels were strongly associated with asthma exacerbations in those children [144,145,146]. Meteorological factors are important modulators in asthmatic children and adults [147,148]. During the COVID-19 pandemic, the most important factors that reduced asthma attacks were the decreased exposure to environmental triggers (e.g., the time spent at home) and the increased adherence to treatment [56,149]. Thus, identifying environmental factors associated with asthma exacerbations could lead to prompt pharmacological interventions [143,150] and offer the possibility of reducing exposure to specific triggers [151,152].

Air pollution is another crucial risk factor for children living in urban areas [153,154,155,156,157,158,159]. In the study by Zhang et al., who examined 17,227 pediatric asthma admissions during the 2015–2016 period in Chinese urban areas, a strong relationship emerged between hospital visits and nitrogen dioxide (NO_2_) ozone (O_3_), and particulate matter of at least 2.5 mm (PM_2.5_) levels [160]. A similar study, also from China, confirmed that PM_2.5_, sulfur dioxide (SO_2_), and NO_2_ atmospheric concentrations were significantly associated with asthma attacks [161]. The effects of SO_2_ were more potent in the cold season and those of NO_2_ during the warm months, while preschool children were more susceptible to increased SO_2_ levels [161]. In the same line, a relevant meta-analysis concluded that NO_2_, SO_2_, and PM_2.5_ levels predispose to future asthma attacks in both children and adults [162]. Interestingly, even short-term exposure to high concentrations of air pollutants may significantly increase the risk of asthma exacerbations [163]. Short-term exposure is associated with reduced interferon beta (IFN-b) expression in the airway epithelium, facilitating viral replication [164,165,166]. Tobacco smoke exposure, either first- or second-hand, has similar effects and may also trigger severe exacerbations [167]. In a prospective study of asthmatic Thai children, daily PM_2.5_ exposure to levels above 12 mcg/m^3^ was associated with asthma exacerbation within the next three days [168]. Accumulating evidence suggests that long-term exposure to air pollution, especially traffic-related air pollution (TRAP), can contribute to new-onset asthma in children and adults [169,170,171]. Four main mechanisms have been described: oxidative stress damage, airway remodeling, the activation of inflammatory pathways and immunological responses, and the enhancement of respiratory sensitization to aeroallergens [171].

Improving the air quality to prevent future asthma exacerbations and new cases of asthma in children would require solid governmental efforts. Until then, the continuous monitoring and online availability of air pollutant concentration and relevant meteorological data should be considered [172,173,174]. Informatics and wearable sensor technologies may further assist in collecting biometric data to understand pediatric asthma triggers and design appropriate and personalized monitoring and prevention strategies [163,175].

## 9. Risk Scores

Admittedly, a single marker for assessing the risk of asthma exacerbations is challenging to identify. Thus, current research focuses on combining risk factors into composite scores using advanced analytic methods, such as machine learning, to improve the risk stratification and recognition of the most vulnerable children [176,177]. These approaches are based on the systematic monitoring of known clinical and lung function exacerbation predictors, also offering the possibility of including widely available biomarkers (e.g., EOS, FeNO) or even air pollutant concentrations and relevant meteorological data [177].

A multidisciplinary, multi-factorial, and personalized approach is mandatory when managing pediatric asthma [13,178,179]. Current guidelines focus on the stepwise escalation/de-escalation of drug therapy to achieve improved control and reduce the risk of exacerbations. Therefore, the prompt identification of symptoms and the longitudinal monitoring of physiologic parameters (including lung function) are important. Huffaker et al. applied the passive nocturnal monitoring of heart rate, respiratory rate, and body movements by using a contactless bed sensor in a small cohort of asthmatic children (n = 16). Asthma symptoms and ACT scores were reported every two weeks. The investigators reported that nocturnal physiologic changes correlated well with asthma symptoms, suggesting that nocturnal physiologic monitoring could represent an objective tool for assessing disease control and predicting asthma exacerbations [180]. In a big cohort of 28,196 patients, Hatoun et al. recognized ten potential predictors that were subsequently included in an asthma exacerbation risk (AER) score [181]. The AER score is calculated monthly by healthcare professionals to identify children at risk for asthma exacerbation within the following year [181]. Another score, the test for respiratory and asthma control in kids (TRACK), has been designed to apply in preschoolers with acute wheezing episodes within the first five days of the event [182]. It has been reported that TRACK predicts a subsequent severe exacerbation (emergency department visit and/or need of systemic corticosteroids) within the next three months; for each 10-unit decrease in TRACK, the probability of a future exacerbation increases by 38% [182].

An advanced monitoring tool, the myAirCoach system, which includes an inhaler adapter, an indoor air-quality monitor, a physical activity tracker, a portable spirometer, a personal FeNO device, and a dedicated smartphone app, has been shown to improve asthma control and the quality of life of asthmatic patients [183,184]. The Biomedical Real-Time Health Evaluation (BREATH) platform is a similar tool that focuses on pediatric patients [185]. Although much work remains to be carried out about measurement collection and standardization, analyzing these data series using machine learning algorithms holds promise for developing reliable personalized predictive tools [186,187,188,189].

## 10. Conclusions

**Figure 1:**
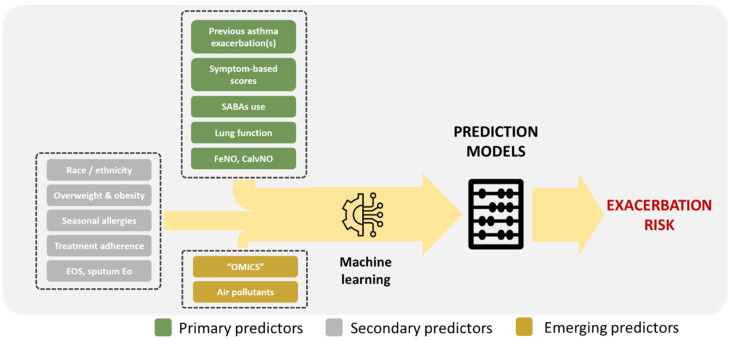
General approach for the prediction of asthma exacerbations in children.

Pediatric asthma is a multifactorial, complex, and dynamic disease, and as such, it cannot be monitored using classical clinical tools or simple biomarkers. The ideal method for predicting the loss of disease control and imminent asthma exacerbations should be based on the combination of patient data (e.g., demographics, symptom-based scores, etc.), lung function measurements, various Th2 inflammation biomarkers (e.g., EOS, FeNO, “omics”, etc.), and environmental exposures (e.g., aeroallergen and air pollutant concentrations, meteorological data, etc.). Machine or deep learning techniques should be used to analyze these big-data series further and ensure reliable and personalized predictions in the context of different disease subtypes. The above approach is summarized in Figure 1. Standardizing the criteria to diagnose asthma exacerbation is equally critical; both loose and stringent definitions of asthma attacks may lead to false associations, thus impeding the generalizability of the prediction models. Finally, an important future aim should be establishing an international pediatric exacerbation network that would significantly facilitate data collection and comparison, as well as assessing innovative technologies and applying relevant predictive strategies in clinical practice.
